# MicroRNA dynamics during hibernation of the Australian central bearded dragon (*Pogona vitticeps*)

**DOI:** 10.1038/s41598-020-73706-9

**Published:** 2020-10-20

**Authors:** Alexander Capraro, Denis O‘Meally, Shafagh A. Waters, Hardip R. Patel, Arthur Georges, Paul D. Waters

**Affiliations:** 1 School of Biotechnology and Biomolecular Sciences, Faculty of Science, UNSW Sydney, Kensington, NSW 2052 Australia; 2 Institute for Applied Ecology, University of Canberra, Canberra, ACT 2601 Australia; 3 School of Women’s & Children’s Health, Faculty of Medicine, UNSW Sydney, Kensington, NSW 2052 Australia; 4 John Curtin School of Medical Research, Australian National University, Canberra, ACT Australia; 5 Present Address: Center for Gene Therapy, Beckman Research Institute of the City of Hope, Duarte, CA 91010 USA

**Keywords:** Computational biology and bioinformatics, Gene ontology, Gene regulatory networks, Sequence annotation, Genetics, Epigenetics, Epigenomics, Gene expression, Gene regulation, Sequencing, Epigenetics, Transcriptomics, Non-coding RNAs, miRNAs, Small RNAs

## Abstract

Hibernation is a physiological state employed by many animals that are exposed to limited food and adverse winter conditions. Controlling tissue-specific and organism wide changes in metabolism and cellular function requires precise regulation of gene expression, including by microRNAs (miRNAs). Here we profile miRNA expression in the central bearded dragon (*Pogona vitticeps*) using small RNA sequencing of brain, heart, and skeletal muscle from individuals in late hibernation and four days post-arousal. A total of 1295 miRNAs were identified in the central bearded dragon genome; 664 of which were novel to central bearded dragon. We identified differentially expressed miRNAs (DEmiRs) in all tissues and correlated mRNA expression with known and predicted target mRNAs. Functional analysis of DEmiR targets revealed an enrichment of differentially expressed mRNA targets involved in metabolic processes. However, we failed to reveal biologically relevant tissue-specific processes subjected to miRNA-mediated regulation in heart and skeletal muscle. In brain, neuroprotective pathways were identified as potential targets regulated by miRNAs. Our data suggests that miRNAs are necessary for modulating the shift in cellular metabolism during hibernation and regulating neuroprotection in the brain. This study is the first of its kind in a hibernating reptile and provides key insight into this ephemeral phenotype.

## Introduction

Animals that hibernate undergo remarkable seasonal change that involves profound modifications in their physiology, morphology and behaviour. Despite general differences amongst species, the adaptive strategies are common to most known hibernators. Typically, hibernation leads to a drastic reduction in basal metabolic activity, oxygen consumption, heart rate and core body temperature. Active reduction in metabolic rate and the lowering of body temperature globally reduces rates of macromolecule synthesis and degradation, to redirect energy expenditure towards management of physiological stress (reviewed in^1^).

Hibernators reprioritise cellular fuel sources, switching from glucose-based sources to triglycerides and fatty acids that are stored prior to hibernation^2^. Stored fats are catabolised via lipolysis, beta oxidation, and ketogenesis. In some cases, such as in hibernating reptiles like the Australian central bearded dragon (*Pogona vitticeps*), large amounts of stored glycogen (in the tail) are used in conjunction with stored triglycerides^3^. Hibernators must induce stress responses to mitigate physiological stress, including nutrient deficiency, compromised immune system, and oxidative- and cold-stress, that would otherwise be lethal. Tissue-specific responses must also be induced to prevent the progression of excitotoxicity in the brain^4^ and muscle atrophy in skeletal muscle^5^.

Studies aiming to understand the molecular architecture of hibernation phenotype have demonstrated control at multiple levels of gene regulation: including epigenetic changes (DNA methylation and histone modification), gene silencing by microRNAs (miRNAs), and protein modifications^6,7^. Amongst these, the role of miRNAs in response to stress, such as during hibernation, is becoming increasingly evident^8–17^.

MicroRNAs are small non-coding RNA molecules ranging from 18 to 22 nucleotides that post-transcriptionally regulate gene expression. miRNAs are initially transcribed as primary miRNAs (pri-miRNAs) with unique secondary hairpin structures that are processed in the nucleus by the RNase Drosha into precursor miRNAs (pre-miRNAs). Pre-miRNAs are further processed, by the RNase Dicer in the cytoplasm, into functional mature miRNA (reviewed in^18^).

Mature miRNA sequences are often conserved within Metazoa with a substitution rate of 3.5%, half of that of 18S ribosomal DNA (7.3%)^19,20^. In humans, miRNAs are known to target and regulate over 60% of protein-coding genes^21^. The 5′ seed region of miRNAs (nt 2–8) complement with 3′-untranslated regions (UTRs) of target mRNAs^22^, recruiting the RNA-induced silencing complex (RISC), which subsequently cleaves and degrades the mRNA transcript^18^. Furthermore, RISC is able to repress translation of mRNAs, without degradation, by modulating the binding of ribosomes and their associated proteins^23^. miRNA-mediated translational repression is potentially a rapid and energy efficient means of regulating gene expression during hibernation that may be particularly important for ‘kickstarting’ normal function after arousal^7^.

In hibernating mammals there are changes in miRNA expression have a clear role in regulating shifts in metabolism^12,13^, resistance to atrophy of skeletal muscle^9,17^ and increased neuroprotection in brain^10^. However, changes in miRNA dynamics remains obscure in hibernators outside of mammals. The central bearded dragon (*Pogona vitticeps*) is a well-established model to study reptilian hibernation as its genome is sequenced^24^, and hibernation can be induced in captivity by reducing temperatures to that experienced during winter. Bearded dragons hibernate during the coldest months of the year (between May to September), where temperatures range from 5 to 18 °C, by burying themselves under soil or seeking refuge in fallen logs or tree stumps^25^. To date, no studies have investigated the natural physiology of bearded dragon hibernation. In captivity, hibernation typically occurs uninterrupted, with minimal loss of body weight and muscle mass. Using mRNA sequencing, we found that pathways involved in prevention of atrophy in skeletal muscle and excitotoxicity in brain were enriched during hibernation^7^.

Here we explored the small RNA profiles of six Australian central bearded dragons in matched brain, heart, and skeletal muscle at two time points; (1) late hibernation, and (2) two months post-arousal from hibernation. We identified miRNAs conserved with *Anolis carolinensis*; the closest related reptile with annotated miRNAs, and *Homo sapiens*, and discovered novel miRNAs in central bearded dragons. Differential expressed miRNAs were identified, as were their predicted target mRNAs. miRNA expression was correlated with gene expression^7^ from data matched tissues and time points. This is the first investigation of miRNAs in a hibernating reptile and revealed key novel and conserved miRNAs involved in regulating the switch in cellular fuel sources and neuroprotection in the brain.

## Results

### Prediction of microRNAs in the central bearded dragon

**Figure 1:**
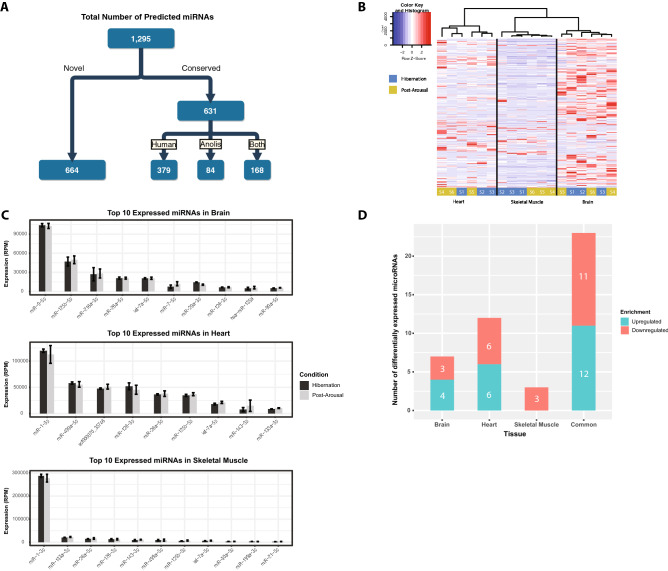
MicroRNA prediction and differential miRNA expression. (**A**) Summary of microRNA prediction performed with miRDeep2. (**B**) Heatmap of expressed miRNAs in all 18 samples with hierarchical clustering. Each column represents a sample and each row a miRNA. The normalized expression of a miRNA (Z-score) within each sample was calculated by subtracting the mean expression across all samples from the sample specific expression value, then dividing by the standard deviation of the mean expression value. Hierarchical clustering and the dendrogram were calculated using Ward’s method. Red Z-scores indicate higher expression and blue lower expression compared to mean expression across all samples. (**C**) Reads per million (RPM) of top 10 expressed microRNAs in post-arousal within brain, heart and skeletal muscle. (**D**) Number of differentially expressed miRNAs between hibernating and post-arousal individuals in brain, heart, skeletal muscle, and combined.

Across all tissues (brain, heart and skeletal muscle), time points (late hibernation and two months post-arousal), and individuals (n = 3 per tissue type at both time points), 1295 miRNAs were predicted with a miRDeep2 score of greater than 4 (equal to signal to noise ratio of 8.1:1) in at least one tissue type (Fig. 1A, Table S1). Of these, we detected 547 that had seed regions conserved with just human miRNAs, and 252 that had seed regions conserved with only anole miRNAs (hereinafter referred to as “conserved miRNAs”) (Fig. 1A). We observed 168 that were conserved with both human and anole. The remaining 664 predicted miRNAs did not share conserved seed regions with either human or anole miRNAs. As such, these miRNAs were considered novel.

### Differential miRNA expression analysis

Differential miRNA expression analysis was performed on all conserved and novel miRNAs. Hierarchical clustering according to expression values of the 1295 predicted miRNAs resulted in distinct groupings of tissue types (Fig. 1B). Brain had the largest number of highly expressed miRNAs, followed by heart, and skeletal muscle. In no tissue was there clear clustering of hibernating or post-arousal individuals (Fig. 1B).

In brain, the highest expressed miRNA during both hibernation and post-arousal was miR-9-5p; an abundant and conserved miRNA in the brain of vertebrates^26,27^ (Fig. 1C). In both heart and skeletal muscle, miR-1-3p (a conserved and critical cardiac and skeletal muscle miRNA^28^) was the most abundant miRNA during hibernation and post-arousal (Fig. 1C).

The greatest number of differentially expressed miRNAs (DEmiRs) between hibernating and aroused animals was observed in heart, followed by brain, and then skeletal muscle (Fig. 1D, Table 1, Fig. S1). In heart and brain, 41.6% (5 out of 12) and 42% (3 out of 7) of DEmiRs were conserved with either human or anole miRNAs, whereas conserved DEmiRs were at 66.6% (2 out of 3) for skeletal muscle.

**Table 1:** List of all differentially expressed microRNAs with FDR < 0.05.

miRNA ID	miRNA	Log_2_ fold change	Adjusted *p* value	Synteny	Number of predicted targets	Differential expression of targets
Brain						
scf000152_19859	Novel	6.527190041	0.02005973	N/A	402	↑ 88 ↓ 102
scf000119_5395	Novel	5.589015217	0.01114128	N/A	24	
scf000549_15459	miR-4696	3.512815814	0.01641398	No	139	
scf000258_20371	miR-1468-5p	1.393059002	0.00210684	No	73	
scf000478_4389	Novel	− 1.122669432	0.00193561	N/A	176	↑ 207 ↓ 286
scf000260_12856	miR-149-3p	− 2.546853144	0.01901295	No	902	
scf000063_9938	Novel	− 4.24394942	0.01805405	N/A	899	
Heart						
scf000119_5395	Novel	5.783988628	0.00789498	N/A	24	↑ 108 ↓ 95
scf000314_20127	mir-203-3p	4.054558206	0.02801801	Yes	66	
scf000714_4624	Novel	3.682805565	0.02694264	N/A	263	
scf000019_10057	miR-338-3p	2.258131288	0.00789498	Yes	280	
scf000415_13898	miR-551-3p	1.959062694	0.02205359	Yes	114	
scf000258_20371	miR-1468-5p	1.544402243	0.00361614	No	73	
scf000052_33382	Novel	− 1.438517169	0.04137763	N/A	252	↑ 209 ↓ 168
scf000478_4389	Novel	− 1.584299915	3.35E−06	N/A	176	
scf001197_18393	Novel	− 1.620271434	3.35E−06	N/A	126	
scf000567_33894	mir-196-5p	− 2.620934423	0.02694264	Yes	198	
scf000172_9618	Novel	− 5.670460842	0.02350143	N/A	53	
scf000433_24590	Novel	− 5.923457527	0.02694264	N/A	148	
Skeletal muscle						
scf000194_40008	mir-460b-5p	− 2.673240877	0.02371199	Yes	451	↑ 135 ↓ 118
scf001197_18393	Novel	− 1.324951293	0.02444514	N/A	239	
scf000568_104	mir-1306-3p	− 0.995901032	0.00029401	Yes	232	
Common						
scf000777_10824	miR-5436-3p	6.064408385	0.0135961	No	129	↑ 102 ↓ 89
scf000094_1910	Novel	5.358012186	0.006831	N/A	135	
scf000119_5395	Novel	5.147128271	3.68E−08	N/A	24	
scf000152_19859	Novel	4.950167134	2.48E−05	N/A	402	
scf000121_1222	Novel	3.912932699	0.00915318	N/A	1	
scf000441_19211	Novel	3.897798095	0.00149224	N/A	39	
scf000513_11489	Novel	3.467354853	0.04490014	N/A	42	
scf000714_4624	Novel	3.467344324	1.61E−07	N/A	13	
scf000072_5856	Novel	3.131090872	0.0135961	N/A	68	
scf000549_15459	miR-4696	2.125605064	0.01145064	No	139	
scf000258_20371	miR-1468-5p	1.448028703	0.00017233	No	73	
scf000739_36870	Novel	− 1.212623676	0.01292055	N/A	45	↑ 271 ↓ 255
scf000478_4389	Novel	− 1.226483643	0.00149224	N/A	176	
scf000030_21650	Novel	− 1.285229809	0.01979265	N/A	402	
scf000260_12856	miR-149-3p	− 1.394757467	0.04167983	No	902	
scf001086_36659	miR-6809-3p	− 2.566189793	0.0077862	No	73	
scf000063_9938	Novel	− 3.310720734	0.00017233	N/A	676	
scf002320_34830	Novel	− 3.320731827	0.00649737	N/A	155	
scf000567_33894	mir-196a-5p	− 3.798661375	0.02233976	Yes	198	
scf000319_14315	Novel	− 4.519654553	0.04450324	N/A	141	
scf000068_14290	miR-7481-3p	− 5.227855526	0.00149224	No	158	
scf000134_8387	Novel	− 6.433907635	0.01292055	N/A	398	
scf000164_27058	Novel	− 6.491719363	0.0009095	N/A	401	

Samples from the three tissues were combined to compare global changes in miRNA expression (hereinafter referred to as common). Twenty-three miRNAs were differentially expressed between hibernating and post-arousal animals (Fig. 1C, Table 1). Six DEmiRs were conserved with human and/or anole. The remaining were novel to bearded dragon.

### Synteny conservation of known differentially expressed miRNAs

For conserved miRNAs, local gene synteny was assessed (Fig. S2A, Table 1). Gene order adjacent to the bearded dragon miRNAs were compared with that of anole, chicken, and human. Both conserved skeletal muscle DEmiRs shared synteny between species, four of five conserved heart DEmiRs shared synteny, whereas no conserved brain DEmiRs shared synteny with any species (Table 1). Only one of six conserved DEmiRs common to all tissues shared synteny with any of the tested species.

### Prediction of miRNA targets

**Figure 2:**
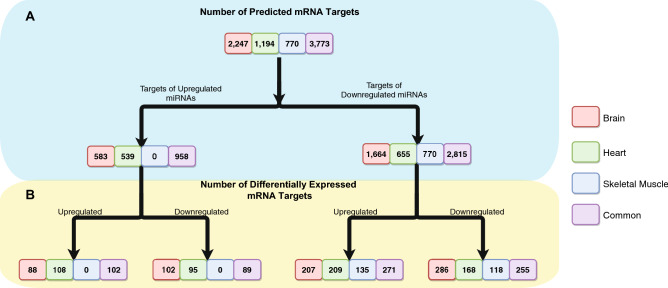
MicroRNA-mRNA target prediction. (**A**) Number of predicted targets of differentially expressed miRNAs in brain, heart, skeletal muscle and combined. (**B)** Number of predicted targets that are differentially expressed within each tissue comparison. Red is brain, green is heart, blue is skeletal muscle, and purple is when all tissues were analysed together (common).

Identification and prediction of target mRNAs was performed on all DEmiRs (Fig. 2A, Fig. S2B). Two tools were used for target prediction of miRNAs; miRanda^29,30^ and RNA22^31^, with target mRNAs predicted by either tool being retained. We predicted 2247 unique targets of the seven DEmiRs in brain (Fig. 2A, Fig. S2B, Table S2), 1194 unique targets of the 12 DEmiRs in heart, and 770 unique targets from the three DEmiR in skeletal muscle. For the 23 novel DEmiRs common to all tissues, 3773 unique targets were identified.

### Differentially expression analysis of target genes

RNA-seq and proteomic data from matched hibernating and post-arousal individuals^7^ was used to assess the expression of DEmiR targets (Table S3). Most target genes were detected in the mRNA expression data, however, only a small proportion of target genes were identified in the proteomic data. Canonically, miRNAs are well known to degrade target mRNAs, therefore, the downregulated targets of upregulated miRNAs during hibernation were of key interest, in addition to the upregulated targets of downregulated miRNAs.

In the brain transcriptome, the upregulated miRNAs during hibernation had 102 out of 583 (17.5%) targets downregulated, and 88 (15.1%) upregulated (Fig. 2B). The downregulated miRNAs in brain had 207 out of 1664 (12.4%) targets upregulated, and 286 (17.2%) downregulated. In the proteome, 41 target genes of upregulated miRNAs were identified, with 3 differentially expressed (1 upregulated and 2 downregulated) (Table S3), including MAP6 which was downregulated in both the transcriptome and proteome. Proteins of 80 target genes of the downregulated miRNAs were identified in brain during hibernation. Five of these proteins were differentially expressed (2 upregulated and 3 downregulated).

In heart, the upregulated miRNAs during hibernation had 95 out of 539 (17.6%) targets were downregulated, and 108 (20.0%) were upregulated (Fig. 2B). The downregulated miRNAs in heart had 209 out of 655 (31.9%) targets upregulated, and 168 (25.6%) downregulated. In the proteome, 16 target genes of upregulated miRNAs were identified; one of which was downregulated (NMRAL1) (Table S3). Proteins of 21 target genes of downregulated miRNAs were identified; 4 of which were differentially expressed (all downregulated).

In skeletal muscle, the downregulated miRNAs during hibernation had 135 out of 770 (17.5%) target genes upregulated and 118 (15.3%) downregulated (Fig. 2B). In the proteome, 9 targets of downregulated miRNAs were successfully identified; 2 of which were differentially expressed (1 upregulated, 1 downregulated) (Table S3).

The upregulated miRNAs common to all tissues had 89 out of 958 (9.3%) target genes downregulated, and 102 (10.6%) upregulated (Fig. 2B, Table S3). The common downregulated miRNAs had 271 out of 2815 (9.6%) were upregulated, and 255 (9.1%) downregulated.

### Functional analysis of differentially expressed targets

To assess potential functional capacity of differentially expressed miRNAs during bearded dragon hibernation, GO enrichment analysis of biological processes was performed on the differentially expressed targets of DEmiRs. Enriched GO terms (*p* value < 0.05) were observed for all tissues (Table S4. However, as most false discovery rates were greater than 0.05 (insignificant), GO enrichment analyses were used as a guide to investigate the predicted biological function of miRNAs.

#### Common to all tissues

In hibernating individuals, upregulated targets of downregulated miRNAs were enriched for 73 diverse biological processes, including: regulation of RNA polymerase II (GO:0006357), chromatin organization (GO:0006325), histone modification (GO:0016570), protein modification by small proteins (GO:0070647), and response to light stimulus (GO:0009416) (Table S4). Three miRNAs that target key glucose and fat metabolism genes were downregulated during hibernation across all three tissues: scf000030_21650, miR-149-3p (scf000260_12856), and scf002320_34830. The targets of these genes include *PPARGC1A*, *CRTC1*, *CRTC2*, and *PRKAG1*. These genes were upregulated during hibernation in all tissues with the exception of *PPARGC1A*, which was downregulated in brain^7^.

miRNAs that target two genes important in miRNA-mediated translational repression (*AGO3* and *CNOT1*) were downregulated during hibernation. *CNOT1* was targeted by scf000478_4389, while *AGO3* was targeted by four downregulated miRNAs: miR-7481-3p (scf000068_14290), scf000164_27058, scf000478_4389 and miR-196a-5p (scf000567_33894).

#### Brain

During hibernation, downregulated target mRNAs of upregulated miRNAs were enriched for one GO term (GO:2000722) (Table S4). Upregulated targets of downregulated miRNAs were enriched for 48 GO terms. GO terms were related to regulation of gene expression, including protein modification by small protein, gene silencing by RNA, negative regulation of gene expression, and histone acetylation (Table S4).

The novel bearded dragon miRNA scf000152_19859 was a particularly interesting DEmiR. It was expressed in hibernators, but undetectable in post-arousal brain. GO term analysis of all target genes of scf000152_19859 revealed enrichment for several neuronal and synaptic signalling processes; including regulation of AMPA receptor activity, regulation of short-term neuronal synaptic plasticity, and regulation of positive synaptic transmission (Table S4). Two targets of this miRNA are *MAP6* and *PLEC*, which were both downregulated at the protein level. *MAP6* mRNA was also downregulated. These two genes are involved in stabilization of microtubules^32,33^. Furthermore, scf000152_19859 targets *CAMK2A* which was downregulated during hibernation at the mRNA level. *GRIN1* mRNA, which is targeted by miR-1468-5p (scf000258_20371), was also downregulated.

#### Heart and skeletal muscle

During hibernation in heart, the downregulated targets of upregulated miRNAs were enriched for one GO term (GO:0030947) (Table S4). In heart, upregulated targets of downregulated miRNAs were enriched for 3 biological processes, including muscle structure development (GO:0061061), and regulation of neurogenesis (GO:0050767). In skeletal muscle of hibernating individuals, upregulated targets of the downregulated miRNA were enriched 26 biological processes. GO terms were related to cellular metabolism, including regulation of metabolic processes (GO:0080090) and response to cold (GO:0070417) (Table S4).

## Discussion

The drastic changes in cellular physiology during hibernation necessitates the need for precise control of gene expression. In mammals, there is increasing evidence for the importance of miRNAs in maintaining correct gene product abundance during hibernation^8–15^. However, the role of miRNAs in reptilian hibernators has yet to be examined. For the first time, we have identified conserved and novel miRNAs in the central bearded dragon genome. A subset of differentially expressed miRNAs correlate with expression of predicted mRNA target during hibernation, particularly involved in cellular metabolism and neuroprotection in brain. Our results support the idea that multi-level regulation of gene expression is required for modulating hibernation and elucidates specific processes that miRNAs modulate during bearded dragon hibernation.

In bearded dragon, differential expression of miRNAs appears to be largely tissue-specific. However, 23 miRNAs were identified as differentially expressed when all tissues were compared between the two time points. Considering the drastic changes in mRNA expression^7^, a surprisingly small number of miRNAs displayed differential expression. However, miRNAs can target multiple mRNAs^34^, so hibernation may only require modulation of a few critical miRNAs. Half of the miRNAs were not annotated in any other species.

### miRNA expression during hibernation correlates with shifts in cellular metabolism

**Figure 3:**
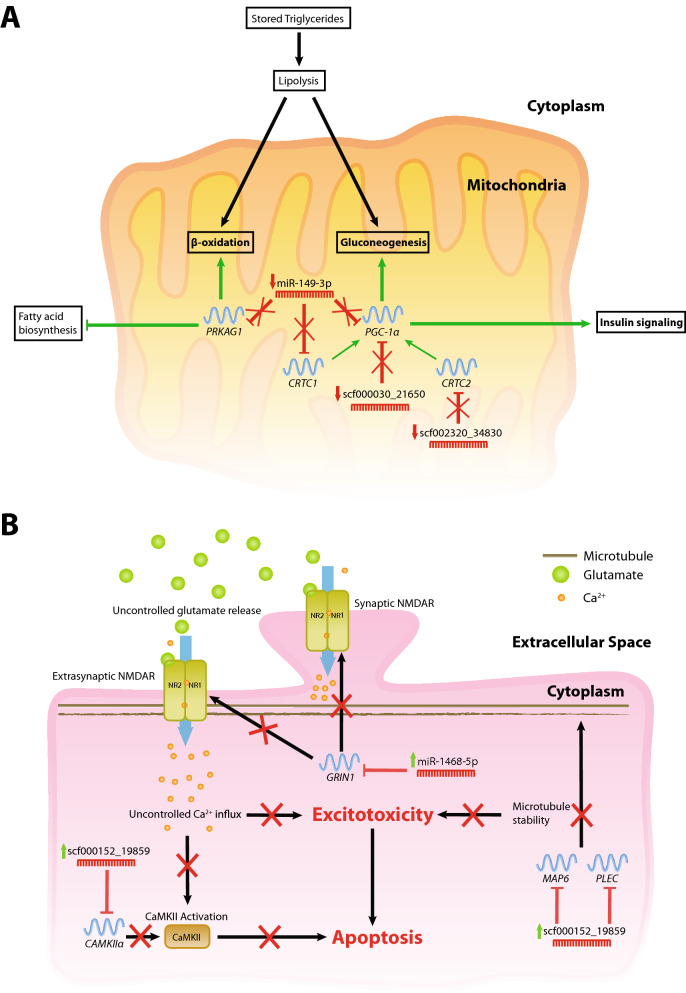
Summary of biological processes under regulation of miRNAs during central bearded dragon hibernation. (**A**) Cellular metabolic processes common to all tissues assessed. (**B**) Cellular processes involved in the progression of excitotoxicity in neurons of brain. miRNAs with green arrows are upregulated during hibernation, with their target mRNAs downregulated. miRNAs shown with red arrows are downregulated during hibernation, with their target mRNAs upregulated. Red crosses refer to active regulation of the process during hibernation.

Insulin resistance is a hallmark of mammalian hibernation (reviewed in^35^). It was proposed that insulin resistance occurs prior to hibernation as a mechanism to store excess body fat, which is subsequently reversed during hibernation. During hibernation the upregulated targets included several key glucose and fat metabolism regulators such as: *PPARGC1A*, *CRTC1*, *CRTC2*, and *PRKAG1* (Fig. 3A). Two downregulated miRNAs were predicted to target *PPARGC1A*, which encodes PGC-1α, a protein critical in regulating energy metabolism and mitochondrial biogenesis. Activation rescues insulin signalling in insulin-resistant mice and induces gluconeogenesis (the production of glucose from non-carbohydrate sources—reviewed in^36^). CRTC1, CRTC2 and PRKAG1; the regulatory subunit of AMPK, are all coactivators of PCG-1α. During energy depletion or stress, AMPK is activated and, together with PGC-1α, activates fatty acid oxidation and increased mitochondrial activity (reviewed in^37^)^38,39^. miR-149-3p (scf000260_12856) targets *PPARGC1A* and *PRKAG1*. During hibernation the reduced expression of miRNAs that target these key metabolic genes may release gene repression to promote increased fatty acid oxidation and gluconeogenesis (Fig. 3A).

### miRNAs facilitate neuroprotective mechanisms in the brain during hibernation

Intracellular Ca^2+^ concentration and homeostasis are essential to proper neurotransmission in brain, with Ca^2+^ signalling regulating functions including synaptogenesis, neuronal plasticity and cell survival of neurons^40^. Dysregulation of Ca^2+^ homeostasis and signalling, a process that occurs in many neurodegenerative diseases, can trigger cell death pathways, such as those regulated by caspases^40–42^. During cerebral stress, a large source of unregulated intake of Ca^2+^ is mediated by synaptic and extrasynaptic *N*-methyl-methyl-D-aspartate receptors (NMDAR) overactivated by excess glutamate release. This can cause major neurological damage via induction of excitotoxicity^43^.

Calcium binding proteins (CaBPs), which act to buffer intracellular Ca^2+^ concentrations^44,45^, and calcium sensor proteins, such as calmodulin (CaM), are crucial mediators Ca^2+^ homeostasis and signalling^46^. When bound to Ca^2+^, CaM can bind and activate key signalling proteins such as CaM-dependant kinase II (CaMKII). In hibernating frogs and hedgehogs cerebellums, CaM and CaBP immunoreactivity decreased during hibernation, respectively, with CaM3 (*CALM3*), calbindin (*CALB1*) and calretinin (*CALB2*) mRNA levels decreasing in the cortex hibernating ground squirrels compared to active squirrels^47^.

A novel bearded dragon miRNA (scf000152_19859) may be particularly important for regulating this process in hibernating individuals (Fig. 3B). scf000152_19859 was upregulated in the brain during hibernation and predicted to target *CAMK2A. CAMK2A*, which encodes for the neuronal-specific isoform of CaMKII; CaMKIIα, was downregulated in the brain during hibernation in bearded dragon^7^. In excitotoxic neurons, CaMKII is persistently activated and initiates progression of apoptosis^48,49^. Inhibition of CaMKIIα activity both prior to and after excitotoxic insult is extremely neuroprotective to rat neuronal cultures, with overexpression significantly increasing neuronal death^50,51^. Our results suggest that the downregulation of CaMKIIα via miRNA activity may be important in prevention of apoptosis progression in the brain of hibernation bearded dragons.

Calcium homeostasis has been implicated in regulating neuroarchitecture, potentially by modulating microtubules and microtubule-associated proteins (MAP), such as tau (MAPT)^41^. MAPT and other MAPs are essential for anchoring NMDARs to the cell membrane^52^. In the brain of hibernating bearded dragons tau protein kinases were upregulated^7^. Significant increases in MAPT phosphorylation has been observed in both mammalian and mollusc hibernation^4,53–55^. Increased phosphorylation of tau protein, such as by CAMKs, reduces its affinity for microtubules (reviewed in^56^), destabilizing the microtubule, and potentially causing disruption of NMDAR anchoring. Furthermore, the NMDAR NR1 subunit *GRIN1* is downregulated in the brain of hibernating bearded dragons. miR-1468-5p, which was upregulated during hibernation, was predicted to target *GRIN1*. The reduction in NMDAR signalling and anchoring to microtubules has been proposed to be a neuroprotective mechanism in hibernating mammals by way of preventing uncontrolled Ca^2+^ release and progression of excitotoxicity^4^.

In addition to targeting *CAMK2A,* scf000152_19859 was predicted to target transcripts of the microtubule-associated proteins MAP6 and plectin (PLEC). Accordingly, mRNA and protein levels of MAP6, and protein levels of PLEC, were downregulated (Table S3)^7^. MAP6 was shown to stabilise microtubules at cold temperatures (below 20 °C), where its absence causes rapid depolymerisation of microtubules in HeLa and mouse embryonic fibroblast cells^32^. The two-fold reduction in MAP6 expression may result in microtubule depolymerisation in order to disrupt anchoring NMDARs in the brain during hibernation. PLEC is a very large protein that links actin, microtubules and intermediate filaments together^33^. Reduced abundance of plectin, with the addition of MAP6, may result in the further destabilisation of microtubules (Fig. 3B).

These observations suggest miRNA-mediated gene expression helps protect against uncontrolled Ca^2+^ influx and induction of excitotoxicity in the brains of hibernating bearded dragons. Furthermore, changes in microtubule dynamics appear to mirror tauopathies (neurodegenerative disorders associated with aggregation of abnormal tau protein), including Alzheimer’s and Parkinson’s disease^57,58^. While MAPT phosphorylation is reversible in hibernators, the similar physiology in tauopathies and hibernation may suggest a common theme^4^. In both cases, destabilization and reduced NMDAR signalling may be a neuroprotective process that reduces the potential for excitotoxicity. As such, much like mammalian hibernators^53,59^, the hibernating brain of bearded dragons could possibly be used as a model for neurological diseases, such as Alzheimer’s and Parkinson’s.

### miRNAs target key translational repression genes

miRNA-mediated translational repression is thought to be vital to regulating the expression of proteins during hibernation^8,60^. In bearded dragon, *AGO3* (the catalytic subunit of non-cleaving RISC), three key CCR4-NOT genes (necessary for RISC-mediated translational repression) and *EIF4ENIF1* (a critical initiation factor for CCR4-NOT) were upregulated during hibernation^7^. Here we observed that four miRNAs targeting *AGO3* and *CNOT1* were each downregulated during hibernation (Table 1, Table S3). This suggests that miRNAs may self-regulate the key genes involved in miRNA-mediated translational repression.

## Conclusion

This study is the first small RNA profiling analysis of a reptile during hibernation and post-arousal. We identified conserved and novel miRNAs in the bearded dragon genome. Differentially expressed miRNAs that target key genes involved in cellular metabolism were uncovered, suggesting that miRNAs play a central role in regulating the phenotype of hibernating bearded dragons. Furthermore, the tissue-specific expression of miRNAs in the brain implies a role in regulating the expression of genes important for neuroprotection. Overall, this study reinforces the importance of miRNAs in regulating adaptive phenotypes, such as hibernation, and elucidates mechanisms that may be vital for survival during hibernation in the central bearded dragon.

## Methods

### Animals and tissue collection

Central bearded dragons (*Pogona vitticeps*) were captive bred and housed at the University of Canberra under a protocol approved by the University of Canberra Animal Ethics Committee (CEAE17-08) and ACT Government License to Keep (K9640). Husbandry practices fulfil the Australian Code for the Care and Use of Animals for Scientific Purposes 8th edition (2013) Sects. 3.2.13–3.2.23.

Captive conditions are as described in Capraro *et al*.^7^. All hibernation individuals are matched with that in Capraro *et al*.^7^, while the post-arousal individuals are not. Commercial sources of vegetables, mice and live insects (crickets and cockroaches) were provided as food, with water available ad libitum. Cages were cleaned thoroughly monthly, with superficial cleaning done daily (removal of faecal matter and unused food, maintenance of clean water containers). Logs and small branches were provided as basking perches and cardboard boxes provided as retreats. Enclosures were lit by a fluorescent lamp, a strong UVB light source, and a floodlamp (as a heat source) on a variable light:dark (L:D) cycle: August–mid-June (13hL:11hD; 22 °C), late June (2 weeks-6hL: 18hD; 18 °C) and winter hibernation (0hL:24hD; 12 °C). For two weeks prior to hibernation, heat and light were reduced and animals were not fed. All heat and UV lights were turned off for 8 weeks and the facility temperature maintained at 12 °C using thermostat control, which stimulated any animals remaining active to hibernate. The conditions of artificial hibernation are chosen to mimic those that occur during natural hibernation, in that ambient temperatures are dropped, and light availability reduced. Body temperatures of hibernating animals was the same as ambient temperature (12 °C) due to the lack of access to heat sources. Animals were inspected throughout hibernation. Those that moved during this period were not included in the study. After arousal from hibernation, animals were subject to full summer conditions (13hL:11hD; 22 °C). Body temperatures of animals was at least 22 °C (ambient) with the addition of access to a heat source.

Whole brain, whole heart and femoral skeletal muscle tissue were collected from three individuals at two time points: late hibernation, and four days post-arousal, as described in Capraro *et al*.^7^. Four days post-arousal individuals were aroused after 8 weeks of hibernation, while late hibernation individuals were sampled two weeks prior to artificial arousal of the post-arousal individuals. All lizards were male. Tissues were collected immediately after euthanizing (lethal injection of sodium pentobarbitone 65 mg/kg by caudal venepuncture), snap frozen in liquid nitrogen and stored at − 80 °C until small RNA extraction. All post-arousal animals were sacrificed between zeitgeber time (ZT) 3 and ZT5, where ZT0 is lights on and ZT13 is lights off. Hibernating animals were sacrificed between circadian time (CT) 3 and CT5, where CT0 is the same time of day as ZT0, however, without lights turning on.

### Small RNA preparation and sequencing

Total RNA was extracted from 30 mg of each tissue. Tissue extracts were homogenized using T10 Basic ULTRA-TURRAX® Homogenizer (IKA, Staufen im Breisgau, Germany), and RNA purified using the miRNeasy Mini Kit (QIAGEN, Hilden, Germany) according to the manufacturer’s instructions. RNase-free DNase (QIAGEN, Hilden, Germany) was used to digest DNase on-column. For each sample, 500 ng of high integrity total RNA (RIN > 9) was used for sequencing library construction with the QIAseq miRNA Library Kit (QIAGEN, Hilden, Germany) according to the manufacturer’s instructions. Seventy-five bp single-ended reads were generated on the Illumina NextSeq 500 platform at the Ramaciotti Centre for Genomics (UNSW Sydney, Australia). All sequence data have been submitted to the NCBI sequence read archive under the BioProject ID PRJNA605672^61^. Raw and normalised read counts are available in Table S5.

### Bioinformatics analysis

Raw sequencing reads were analyzed with FastQC (v0.11.5)^62^ and low quality bases were removed using Trim Galore (v0.0.4.4)^63^ with the following options: –phred33 –gzip –length 16 –max_length 24 –adapter AACTGTAGGCACCATCAAT –three_prime_clip_R1 1. miRDeep2 (v2.0.1.2)^64^ was used to map reads to the genome, predict conserved and novel miRNAs, and quantify number of miRNA reads against the central bearded dragon genome^24^ using the following settings: -d -e -h -i -j -l 18 -p -v -n -o 8. Predicted miRNAs were compared to the miRBase databases (Release 22.1)^65^ of known human (*Homo sapiens*) and green anole (*Anolis carolinensis*) miRNAs. miRNAs with a miRDeep2 score of greater than 4 were considered real; conferring to a signal-to-noise ratio of greater than 8.1:1. Sequences of novel miRNAs were searched against the entire miRBase^65^ database to verify that the miRNA does not exist in species other than human and anole. Differential expression analysis of miRNAs was performed with DESeq2 (v1.22.2)^66^. Synteny of bearded dragon miRNAs was determined by comparing the order of up- and downstream genes to that of anole, chicken, and human. Only miRNAs that shared the same up- and downstream genes with either three species were considered syntenous.

All graphs were plotted with R (3.4.2)^67^, RStudio (1.1.383)^68^, and ggplot2 (2.2.1)^69^. miRNAs with a log_2_ fold-change greater than 0.75 and adjusted p-value less than 0.05 were considered differentially expressed. miRanda (v3.3a) (with the options: -sc 150 -en -20 -strict)^29,30^ and RNA22 (v2.0) (with default options)^31^ were used to predict the target binding to mRNAs of the DEmiRs (Table S2).

mRNA-seq and proteomic data was gathered from Capraro *et al. *^7^. Gene ontology (GO) enrichment analysis was performed with GOrilla on differentially expressed target mRNAs using mRNA-seq data (last accessed 2/6/20)^70^. Unranked lists of upregulated and downregulated genes in each condition and tissues were compared to a background list; genes that were expressed (greater than 10 counts per million) within each tissue. All graphs were plotted with R (3.4.2)^67^, RStudio (1.1.383)^68^, and ggplot2 (2.2.1)^69^.

### Ethics approval

Experimentation using animals was approved by the University of Canberra Animal Ethics Committee (CEAE17-08) and are in accordance with ACT Government License to Keep (K9640). Husbandry practices fulfill the Australian Code for the Care and Use of Animals for Scientific Purposes 8th edition (2013) sections 3.2.13–3.2.23.

## Supplementary information

Supplementary Figures.

Supplementary Table S1.

Supplementary Table S2.

Supplementary Table S3.

Supplementary Table S4.

Supplementary Table S5.

Supplementary Legends.

## Data Availability

RNA-seq data are available in the NCBI short read archive under the BioProject ID PRJNA476034 (https://www.ncbi.nlm.nih.gov/bioproject/476034) and miRNA-seq data is available under the BioProject ID PRJNA605672 (https://www.ncbi.nlm.nih.gov/bioproject/605672). Computer code for processing and analyzing sequence and mass spectrometry data is available on request.

## Abbreviations

Ca^2^^+^: Calcium ions
CPM: Counts per million
CT: Circadian time
DEmiR: Differentially expressed miRNA
FDR: False discovery rate
GO: Gene Ontology
MAPT: Microtubule associated protein tau
miRNA: MicroRNA
mRNA-seq: MRNA sequencing
NMDAR: N-methyl-D-aspartate receptor
Pre-miRNA: Precursor microRNA
Pri-miRNA: Primary microRNA
RISC: RNA-induced silencing complex
SUMO: Small ubiquitin-like modifiers
TCA: Tricarboxylic acid
TGF-β: Transforming growth factor beta-receptor
UTR: Untranslated region
ZT: Zeitgeber time

## References

[1] Andrews MT (2007). Advances in molecular biology of hibernation in mammals. BioEssays.

[2] Storey KB, Storey JM (2010). Metabolic rate depression: the biochemistry of mammalian hibernation. Adv. Clin. Chem..

[3] Haggag G, Raheem KA, Khalil F (1966). Hibernation in reptiles—III. Tissue analysis for glycogen and high energy phosphate compounds. Comp. Biochem. Physiol..

[4] Arendt T, Bullmann T (2013). Neuronal plasticity in hibernation and the proposed role of the microtubule-associated protein tau as a “master switch” regulating synaptic gain in neuronal networks. Am. J. Physiol-Reg I.

[5] Cotton CJ (2016). Skeletal muscle mass and composition during mammalian hibernation. J. Exp. Biol..

[6] Storey KB (2015). Regulation of hypometabolism: insights into epigenetic controls. J. Exp. Biol..

[7] Capraro A (2019). Waking the sleeping dragon: gene expression profiling reveals adaptive strategies of the hibernating reptile Pogona vitticeps. BMC Genom..

[8] Dubuc A, Storey KB (2008). Differential expression of microRNA species in organs of hibernating ground squirrels: a role in translational suppression during torpor. BBA-Gene Regul. Mech..

[9] Kornfeld SF, Biggar KK, Storey KB (2012). Differential expression of mature microRNAs involved in muscle maintenance of hibernating little brown bats, Myotis lucifugus: a model of muscle atrophy resistance. Genom. Proteom. Bioinform..

[10] Biggar KK, Storey KB (2014). Identification and expression of microRNA in the brain of hibernating bats, Myotis lucifugus. Gene.

[11] Wu C-W, Biggar KK, Storey KB (2014). Expression profiling and structural characterization of microRNAs in adipose tissues of hibernating ground squirrels. Genom. Proteom. Bioinform..

[12] Yuan L (2015). Down but not out: The role of microRNAs in hibernating bats. PLoS ONE.

[13] Hadj-Moussa H (2016). The hibernating South American marsupial, Dromiciops gliroides, displays torpor-sensitive microRNA expression patterns. Sci. Rep..

[14] Wu C-W, Biggar KK, Luu BE, Szereszewski KE, Storey KB (2016). Analysis of microRNA expression during the torpor-arousal cycle of a mammalian hibernator, the 13-lined ground squirrel. Physiol. Genom..

[15] Liu Y (2010). Genomic analysis of miRNAs in an extreme mammalian hibernator, the Arctic ground squirrel. Physiol. Genom..

[16] Leung AK, Sharp PA (2010). MicroRNA functions in stress responses. Mol. Cell.

[17] Luu BE (2019). MicroRNAs facilitate skeletal muscle maintenance and metabolic suppression in hibernating brown bears. J. Cell. Physiol..

[18] O'Brien J, Hayder H, Zayed Y, Peng C (2018). Overview of microRNA biogenesis, mechanisms of actions, and circulation. Front. Endocrinol..

[19] Wheeler BM (2009). The deep evolution of metazoan microRNAs. Evol. Dev..

[20] Weber MJ (2005). New human and mouse microRNA genes found by homology search. FEBS J..

[21] Friedman RC, Farh KK-H, Burge CB, Bartel DP (2009). Most mammalian mRNAs are conserved targets of microRNAs. Genome Res..

[22] Huang Y (2011). Biological functions of microRNAs: a review. J. Physiol. Biochem..

[23] Wilczynska A, Bushell M (2015). The complexity of miRNA-mediated repression. Cell Death Differ.

[24] Georges A (2015). High-coverage sequencing and annotated assembly of the genome of the Australian dragon lizard Pogona vitticeps. GigaScience.

[25] Wells RW (1971). Hibernation—bearded dragons. Herpetofauna.

[26] Sempere LF (2004). Expression profiling of mammalian microRNAs uncovers a subset of brain-expressed microRNAs with possible roles in murine and human neuronal differentiation. Genome Biol..

[27] He M (2012). Cell-type-based analysis of microRNA profiles in the mouse brain. Neuron.

[28] Chen J-F (2006). The role of microRNA-1 and microRNA-133 in skeletal muscle proliferation and differentiation. Nat. Genet..

[29] Betel D, Wilson M, Gabow A, Marks DS, Sander C (2008). The microRNA.org resource: targets and expression. Nucleic Acids Res..

[30] John, B. *et al.* Human microRNA targets. *PLoS Biol.* **2** (2004). 10.1371/journal.pbio.0020363 PMC521178 15502875

[31] Loher P, Rigoutsos I (2012). Interactive exploration of RNA22 microRNA target predictions. Bioinformatics.

[32] Delphin C (2012). MAP6-F is a temperature sensor that directly binds to and protects microtubules from cold-induced depolymerization. J. Biol. Chem..

[33] Svitkina TM, Verkhovsky AB, Borisy GG (1996). Plectin sidearms mediate interaction of intermediate filaments with microtubules and other components of the cytoskeleton. J. Cell Biol..

[34] Zhang F, Wang D (2017). The pattern of microRNA binding site Distribution. Genes.

[35] Martin SL (2008). Mammalian hibernation: a naturally reversible model for insulin resistance in man?. Diab. Vasc. Dis. Res..

[36] Liang H, Ward WF (2006). PGC-1α: a key regulator of energy metabolism. Adv. Physiol. Educ..

[37] Cantó C, Auwerx J (2009). PGC-1alpha, SIRT1 and AMPK, an energy sensing network that controls energy expenditure. Curr. Opin. Lipidol..

[38] Jones RG (2005). AMP-activated protein kinase induces a p53-dependent metabolic checkpoint. Mol. Cell.

[39] Hardie DG, Carling D, Carlson M (1998). The AMP-activated/SNF1 protein kinase subfamily: metabolic sensors of the eukaryotic cell?. Annu. Rev. Biochem..

[40] Zündorf G, Reiser G (2011). Calcium dysregulation and homeostasis of neural calcium in the molecular mechanisms of neurodegenerative diseases provide multiple targets for neuroprotection. Antioxid. Redox Sign..

[41] Mattson MP (1992). Calcium as sculptor and destroyer of neural circuitry. Exp. Gerontol..

[42] Mattson M, Rydel R, Lieberburg I, Smith-Swintosky V (1993). Altered calcium signaling and neuronal injury: stroke and Alzheimer's disease as examples a. Ann. N. Y. Acad. Sci..

[43] Arundine M, Tymianski M (2003). Molecular mechanisms of calcium-dependent neurodegeneration in excitotoxicity. Cell Calcium.

[44] Fairless R, Williams SK, Diem R (2019). Calcium-binding proteins as determinants of central nervous system neuronal vulnerability to disease. Int. J. Mol. Sci..

[45] Schwaller, B. Emerging functions of the “Ca2+ buffers” parvalbumin, calbindin D-28k and calretinin in the brain. *Handbook of neurochemistry and molecular neurobiology: Neural protein metabolism and function*, 197–222 (2007).

[46] Simon B, Huart A-S, Wilmanns M (2015). Molecular mechanisms of protein kinase regulation by calcium/calmodulin. Bioorgan. Med. Chem..

[47] Schwartz C, Hampton M, Andrews MT (2013). Seasonal and regional differences in gene expression in the brain of a hibernating mammal. PLoS ONE.

[48] Rostas JA (2017). Ischaemia-and excitotoxicity-induced CaMKII-Mediated neuronal cell death: the relative roles of CaMKII autophosphorylation at T286 and T253. Neurochem. Int..

[49] Rostas JA, Spratt NJ, Dickson PW, Skelding KA (2017). The role of Ca2+-calmodulin stimulated protein kinase II in ischaemic stroke–A potential target for neuroprotective therapies. Neurochem. Int..

[50] Ashpole NM, Hudmon A (2011). Excitotoxic neuroprotection and vulnerability with CaMKII inhibition. Mol. Cell. Neurosci..

[51] Vest RS, O'Leary H, Coultrap SJ, Kindy MS, Bayer KU (2010). Effective post-insult neuroprotection by a novel Ca2+/calmodulin-dependent protein kinase II (CaMKII) inhibitor. J. Biol. Chem..

[52] Volianskis A (2015). Long-term potentiation and the role of N-methyl-D-aspartate receptors. Brain Res..

[53] Arendt T (2003). Reversible paired helical filament-like phosphorylation of tau is an adaptive process associated with neuronal plasticity in hibernating animals. J. Neurosci..

[54] Su B (2008). Physiological regulation of tau phosphorylation during hibernation. J. Neurochem..

[55] Gattoni G, Bernocchi G (2019). Calcium-binding proteins in the nervous system during hibernation: neuroprotective strategies in hypometabolic conditions?. Int. J. Mol. Sci..

[56] Morris M, Maeda S, Vossel K, Mucke L (2011). The many faces of tau. Neuron.

[57] Pellegrini L, Wetzel A, Grannó S, Heaton G, Harvey K (2017). Back to the tubule: microtubule dynamics in Parkinson’s disease. Cell. Mol. Life Sci..

[58] Crespo-Biel N, Theunis C, Van Leuven F (2012). Protein tau: prime cause of synaptic and neuronal degeneration in Alzheimer's disease. Int. J. Alzheimers Dis..

[59] Zhou F (2001). Hibernation, a model of neuroprotection. Am. J. Clin. Pathol..

[60] Storey KB (2009). Out cold: biochemical regulation of mammalian hibernation–a mini-review. Gerontology.

[61] Leinonen R, Sugawara H, Shumway M, Collaboration INSD (2010). The sequence read archive. Nucleic Acids Res..

[62] Andrews, S. FastQC: A quality control tool for high throughput sequence data [Online]. Available online at: http://www.bioinformatics.babraham.ac.uk/projects/fastqc/.

[63] Krueger, F. Trim galore—a wrapper tool around Cutadapt and FastQC to consistently apply quality and adapter trimming to FastQ files. **516**, 517 (2015).

[64] Kim D, Langmead B, Salzberg SL (2015). HISAT: a fast spliced aligner with low memory requirements. Nat. Methods.

[65] Kozomara A, Birgaoanu M, Griffiths-Jones S (2018). miRBase: from microRNA sequences to function. Nucleic Acids Res..

[66] Love MI, Huber W, Anders S (2014). Moderated estimation of fold change and dispersion for RNA-seq data with DESeq2. Genome Biol..

[67] R: A language and environment for statistical computing. (R Foundation of Statistical Computing, Vienna, Austria, 2010).

[68] RStudio: Integrated Development Environment for R (RStudio, Inc., Boston, MA, 2016).

[69] ggplot2: Elegant Graphics for Data Analysis (Springer-Verlag New York, 2009).

[70] Eden E, Navon R, Steinfeld I, Lipson D, Yakhini Z (2009). GOrilla: a tool for discovery and visualization of enriched GO terms in ranked gene lists. BMC Bioinform..

