# Supplementary material for: MicroRNA dynamics during hibernation of the Australian central bearded dragon (Pogona vitticeps)
Source: Sci Rep. 2020 Oct 20;10:17854. doi: 10.1038/s41598-020-73706-9 (PMC7576210; doi:10.1038/s41598-020-73706-9)
Supplement: Supplementary file 1 — Supplementary Figures. [file 41598_2020_73706_MOESM1_ESM.pdf]

Supplementary Material

MicroRNA dynamics during hibernation of the Australian central bearded dragon (*Pogona vitticeps*)

Alexander Capraro[1,†], Denis O'Meally[2,*], Shafagh A Waters[3], Hardip R Patel[4], Arthur Georges[2], Paul D Waters[1]

1.School of Biotechnology and Biomolecular Sciences, Faculty of Science, UNSW Sydney, NSW 2052, Australia; a.capraro@unsw.edu.au (AC), p.waters@unsw.edu.au (PDW)

2.Institute for Applied Ecology, University of Canberra ACT 2601, Australia; georges@aerg.canberra.edu.au (AG)

3.School of Women's & Children's Health, Faculty of Medicine, UNSW Sydney, NSW 2052, Australia; shafagh.waters@unsw.edu.au (SW)

4.John Curtin School of Medical Research, Australian National University, Canberra, ACT, Australia; hardip.patel@anu.edu.au (HP)

[*]Current address: Center for Gene Therapy, Beckman Research Institute of the City of Hope, Duarte, CA 91010, USA; domeally@coh.org (DOM)

[†]Correspondance: a.capraro@unsw.edu.au

Supplementary Figures

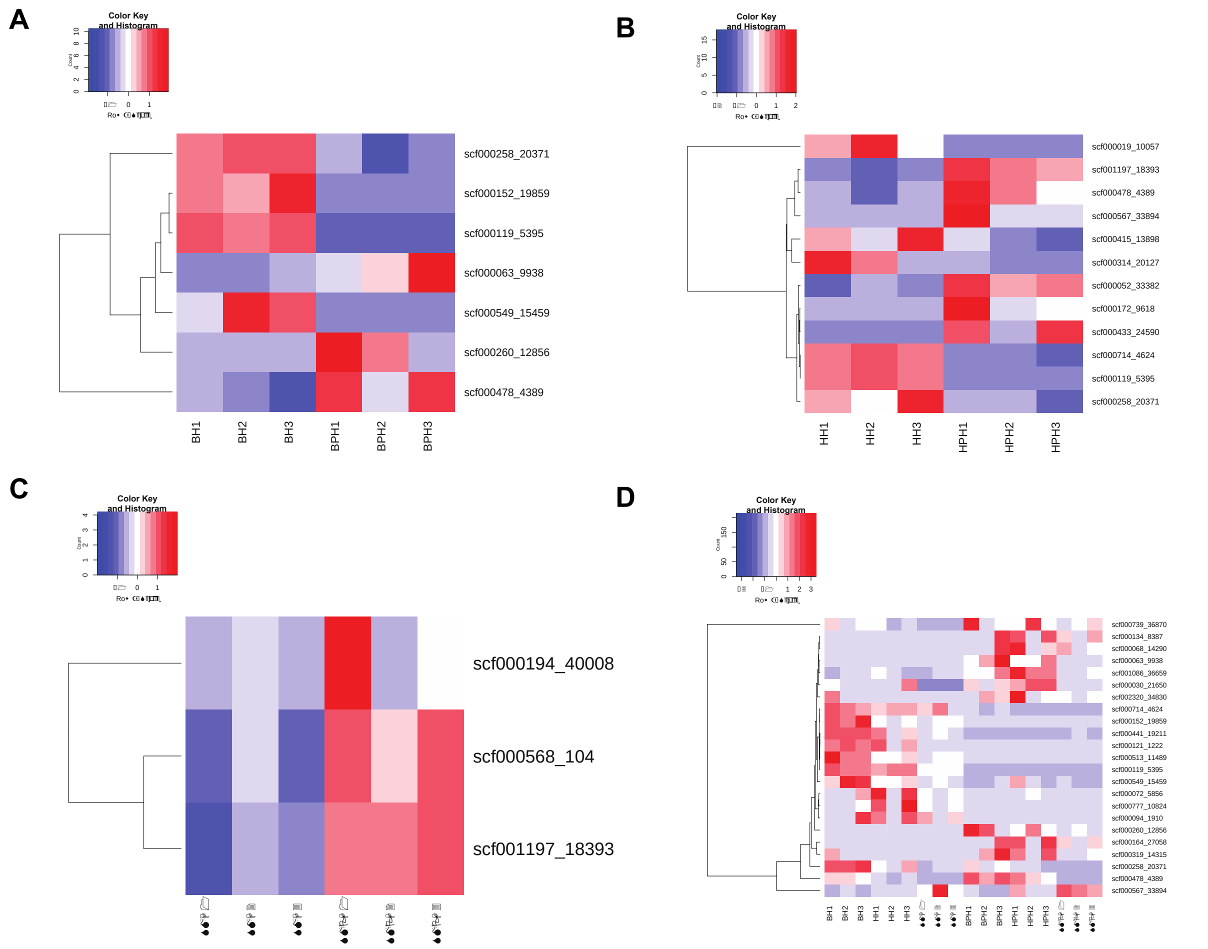

Fig. S1. Differential expression of microRNAs. Heatmap of differentially expressed miRNAs in **A** brain, **B** heart, **C** skeletal muscle, and **D** combined. Each column represents a sample and each row a miRNA. The normalized expression of a miRNA (Z-score) within each condition was calculated by subtracting the mean expression across all samples from the sample specific expression value, then dividing by the standard deviation of the mean expression value. Row hierarchical clustering and the dendrogram were calculated using Ward's method. Red Z-scores indicate higher expression and blue lower expression compared to mean expression across all samples. B = brain, H = heart, SM = skeletal muscle. H = hibernation, PH = post-arousal.

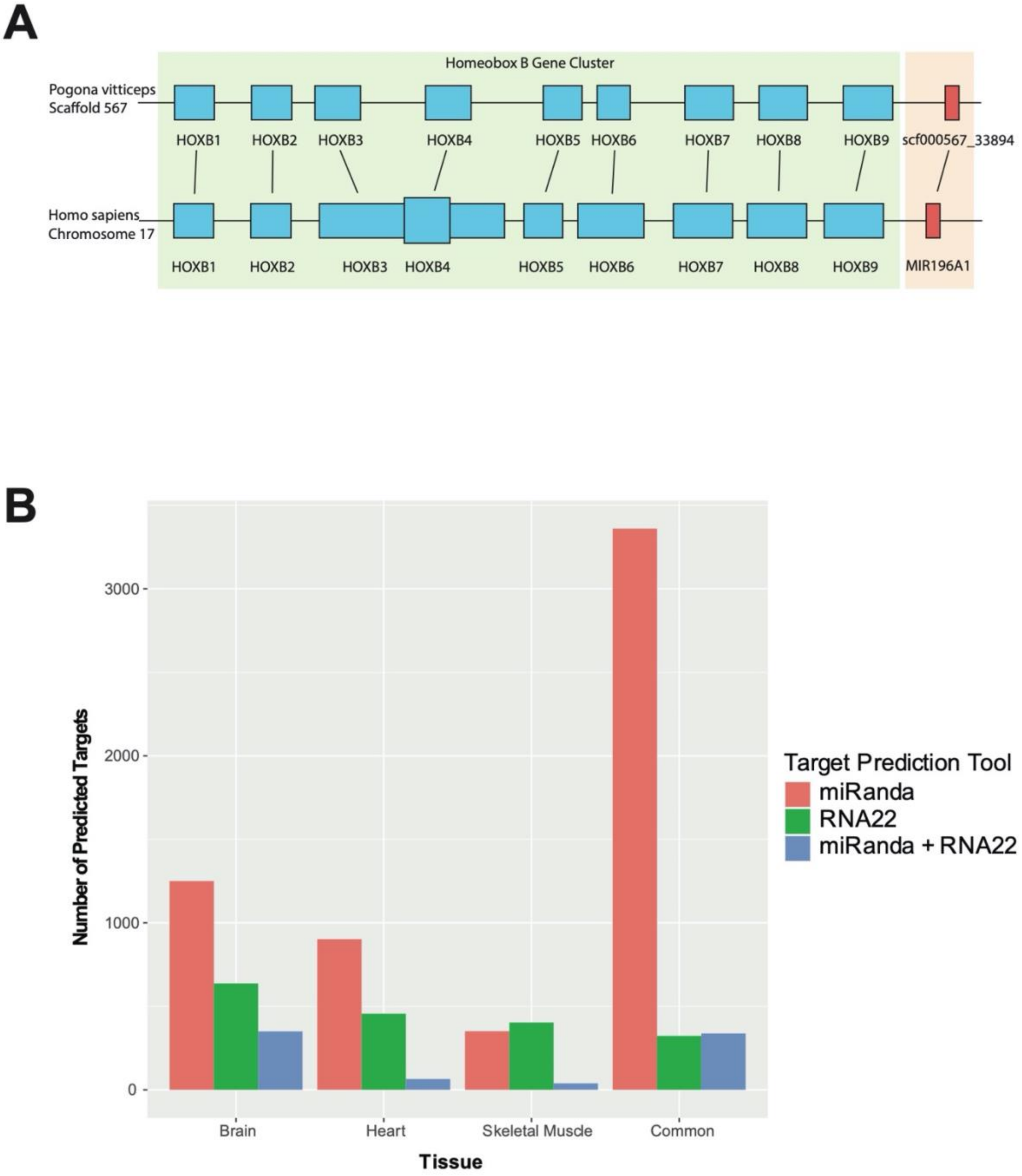

Fig. S2. MicroRNA and mRNA target prediction. A Synteny analysis of mir-196 between *Pogona vitticeps* (scf000567_33894) and *Homo sapiens* (MIR196A1). **B** Number of predicted mRNA targets by multiMiR, miRanda and RNA22.
