# Supplementary material for: MicroRNA dynamics during hibernation of the Australian central bearded dragon (Pogona vitticeps)
Source: Sci Rep. 2020 Oct 20;10:17854. doi: 10.1038/s41598-020-73706-9 (PMC7576210; doi:10.1038/s41598-020-73706-9)
Supplement: Supplementary file 7 — Supplementary Legends. [file 41598_2020_73706_MOESM7_ESM.docx]

**Additional File 1: Table S1. Annotation of microRNAs in the central bearded dragon genome.** Full list of all annotated microRNAs in the central bearded dragon genome as outputted from miRDeep2.

**Additional File 2: Fig. S1. Differential expression of microRNAs.** Heatmap of differentially expressed miRNAs in **A** brain, **B** heart, **C** skeletal muscle, and **D** combined. Each column represents a sample and each row a miRNA. The normalized expression of a miRNA (Z-score) within each condition was calculated by subtracting the mean expression across all samples from the sample specific expression value, then dividing by the standard deviation of the mean expression value. Row hierarchical clustering and the dendrogram were calculated using Ward’s method. Red Z-scores indicate higher expression and blue lower expression compared to mean expression across all samples. B = brain, H = heart, SM = skeletal muscle. H = hibernation, PH = post-arousal.

**Additional File 3: Fig. S2. MicroRNA and mRNA target prediction. A** Synteny analysis of mir-196 between *Pogona vitticeps* (scf000567_33894) and *Homo sapiens* (MIR196A1). **B** Number of predicted mRNA targets by multiMiR, miRanda and RNA22.

**Additional File 4: Table S2. mRNA targets of all differentially expressed microRNAs.** Full list of mRNA targets of all differentially expressed microRNAs. The list contains mRNA targets of conserved miRNAs predicted by multiMiR and targets of novel miRNAs predicted by miRanda and RNA22.

**Additional File 5: Table S3. Differential gene expression of mRNA targets of differentially expressed microRNAs.** Full list of mRNA target expression of differentially expressed miRNAs for brain, heart, skeletal muscle, and common genes as outputted from EdgeR. Log_2_ fold change is relative to hibernation (*i.e.* > 1 Log_2_FC is higher expression during hibernation).

**Additional File 6: Table S4. Gene ontology enrichment analysis data of differentially expressed mRNA targets of differentially expressed microRNAs.** Full list of enriched biological pathway gene ontologies in upregulated and downregulated mRNA target datasets in brain, heart, skeletal muscle, and common genes as outputted from GOrilla.

**Additional File 7: Table S5. Raw and normalised miRNA reads.** Full list of microRNAs predicted by miRDeep2 and the respective raw and normalised expression in each of the 18 samples.
